# *Pseudidotheaarmata* sp. n., a new isopod of the genus *Pseudidothea* (Crustacea, Malacostraca, Isopoda) from the Atlantic sector of the Southern Ocean

**DOI:** 10.3897/BDJ.10.e76864

**Published:** 2022-02-17

**Authors:** Nicholas Francesco Noli, Davide Di Franco, Stefano Schiaparelli, Angelika Brandt

**Affiliations:** 1 Dipartimento di Scienze della Terra dell'Ambiente e della Vita (DISTAV), Università di Genova, Genova, Italy Dipartimento di Scienze della Terra dell'Ambiente e della Vita (DISTAV), Università di Genova Genova Italy; 2 Senckenberg Research Institute and Natural History Museum, Frankfurt am Main, Germany Senckenberg Research Institute and Natural History Museum Frankfurt am Main Germany; 3 Goethe University Frankfurt, Institute for Ecology, Diversity and Evolution, Frankfurt am Main, Germany Goethe University Frankfurt, Institute for Ecology, Diversity and Evolution Frankfurt am Main Germany

**Keywords:** Southern Ocean, morphology, ecology, distribution

## Abstract

**Background:**

In the framework of the British Antarctic Survey (BAS) Expedion JR 15005 SO-AntEco, held in February-March 2016, the South Orkney Islands seafloor was sampled in order to investigate the distribution and composition of benthic communities around the area.

**New information:**

A new species of the genus *Pseudidothea* Ohlin, 1901 is described from the Burdwood Bank area (South Orkney Islands). It has been collected during the SO-AntEco JR15005 RRS James Clark Ross expedition under the lead of the British Antarctic Survey (BAS). The new species, *Pseudidotheaarmata* sp. n., is very similar to *P.scutata* (Stephensen, 1947); however, it is characterised by peculiar supra-ocular spines and a different tubercular pattern. The study of the species of the *Pseudidothea* helps to better understand the diversity of the Pseudidotheidae in the Southern Ocean.

## Introduction

**Figure 1.**
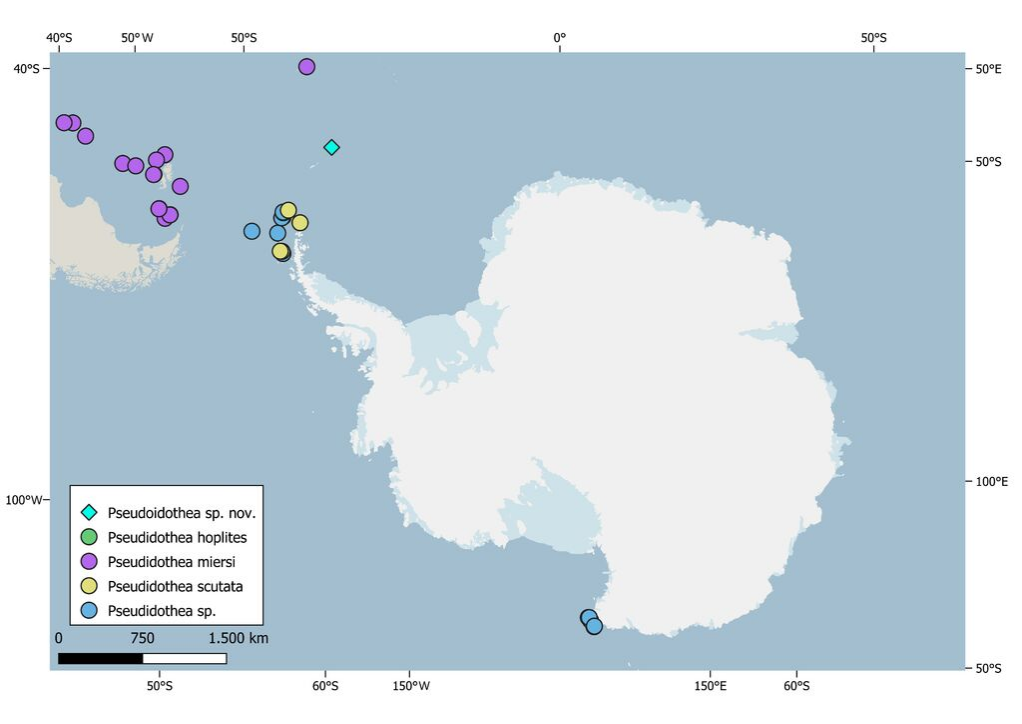
Distribution records of *Pseudidothea* Ohlin, 1901 in the Southern Ocean. Light blue square represents the location of *Pseudidotheaarmata* sp. n, recorded during the SO-AntEco JR15005 RRS James Clark Ross expedition, in Burdwood Bank area (South Orkneys) in the framework of the British Antarctic Survey (BAS), 2016.

The family Pseudidotheidae Ohlin, 1901 is monogeneric with the only genus *Pseudidothea* Ohlin, 1901, comprising four accepted species. The family was erected through the description of its type species *P.bonnieri* Ohlin, 1901, now accepted as *P.miersi* (Studer, 1884) and was recently re-described by Poore and Bardsley (2004). According to WoRMS (World Register of Marine Species) and RAMS (Register of Antarctic Marine Species), only *P.scutata* Stephensen, 1947 (Stephensen 1947) occurs in the Southern Ocean in the area of the Antarctic Peninsula, off Elephant Island and South Shetlands. However, undetermined records of the genus *Pseudidothea* have been reported in several parts of the Antarctic Peninsula and in the Mawson Bank area, Ross Sea (Mills 2020) (Fig. 1).

The biogeographic knowledge of this genus is as follows: in the Southern Hemisphere, most of the records are from Chile (*P.miersi* (Studer 1884, Scarabino et al. 2008)), South Australia (*P.hoplites* (Poore and Bardsley 2004)) and New Zealand (*P.richardsoni* Hurley, 1957 (Hurley 1957)); only one specimen was found in the Southern Ocean in 1927 and has been described by Stephensen (1947) as *Microarcturusscutatus* (Stephensen, 1947). In 1990, it was re-described and placed in the genus Pseudidothea as *P.scutata* by Brandt and Wägele (1990).

In order to improve the biogeographic knowledge of the species of the Southern Ocean, *P.armata* sp. n. is described herein. Though only one specimen is available and used for the description, clear characteristics provide sufficient evidence that the new species differs from the closest morphological (and geographical) species *P.scutata*.

## Materials and methods

### Taxon sampling

The specimen was collected at Burdwood Bank, during the British Antarctic Survey (BAS) Expedition JR 15005 (https://www.bodc.ac.uk/resources/inventories/cruise_inventory/reports/jr15005.pdf) on board the RRS James Clark Ross, by means of a Rauschert dredge at a depth of 852 m (Station 143, 18 April 2016, 60°33.526'S, 41°5.306'W). After the first sorting on board, the specimen was stored and fixed with 96% ethanol in order to preserve it for further genetic analysis.

### Photography and laboratory analyses

Only one specimen, the holotype of the new species, was found during the campaign. The holotype was not dissected in order to preserve it for further studies and it was drawn in dorsal and lateral views following standard descriptions (Wilson 2008). Drawings were performed using a camera lucida, followed by digital inking made by combining the stack photos and the scanned hand-made drawings as layers. The graphic software used was Autodesk SketchBook and digital inking was performed with an XP-PEN Deco 02 graphic tablet. Stacks were obtained by using a Canon EOS 600D and a Leica 125 C, equipped with a Leica DMC 4500 camera. The use of stack photos as the base layer fordigital-inking work is not new for crustacean illustrations (see, for example, the paper by Verheye and D’Udekem D’Acoz (2020)).

### Additional distribution data

Additional distributional data of *Pseudidothea* (Rdmpage 2016, Registry-Migration.Gbif.Org 2016, Mackay 2018, Appiah-Madson and Distel 2019, Data Manager 2019, Mackay 2019, Mills 2020, Registry-Migration.Gbif.Org 2020, Mackay 2021, Orrell and Informatics Office 2021, Registry-Migration.Gbif.Org 2021, Tablado 2021) were provided through GBIF (Global Biodiversity Information Facility, available from https://www.gbif.org/) and OBIS (Ocean Biodiversity Information System https://obis.org/).

Quality check and data cleaning were performed using bibliographic research and the rgbif package (https://CRAN.R-project.org/package=rgbif) in RStudio software.

Maps were drawn using the QGIS (QGIS.org 2021) package QAntarctica (Matsuoka et al. 2018).

### Morphological abbreviations

A = antenna;

P = pereopods;

PL = pleopod;

UR = uropod;

MNA = Italian National Antarctic Museum (Section of Genoa), Genoa;

PNRA = Italian National Antarctic Program;

BAS = British Antarctic Survey

## Taxon treatments

### Pseudidothea armata

Noli, Di Franco, Schiaparelli, Brandt 2021 sp. n.

B267576F-8FC1-58C0-B4F7-4003045B3D75

F4392D41-91CF-458B-ACDE-533BAD913573

#### Materials

- **Type status:** Holotype. **Occurrence:** catalogNumber: MNA 10749; individualCount: 1; sex: male; lifeStage: adult; **Taxon:** kingdom: Animalia; phylum: Arthropoda; class: Malacostraca; order: Isopoda; family: Pseudidotheidae; genus: Pseudidothea; **Location:** continent: Antarctica; locality: Burdwood Bank; verbatimDepth: 852; decimalLatitude: -60.55876; decimalLongitude: -41.08843; **Identification:** identifiedBy: Nicholas Noli; dateIdentified: 2019; **Event:** eventID: St143; samplingProtocol: bottom trawl; year: 2016; month: 3; day: 18; **Record Level:** type: PhysicalObject; basisOfRecord: PreservedSpecimen

#### Description

Measurement. BL = 15 mm; BW = 5 mm.

**Figure 2.**
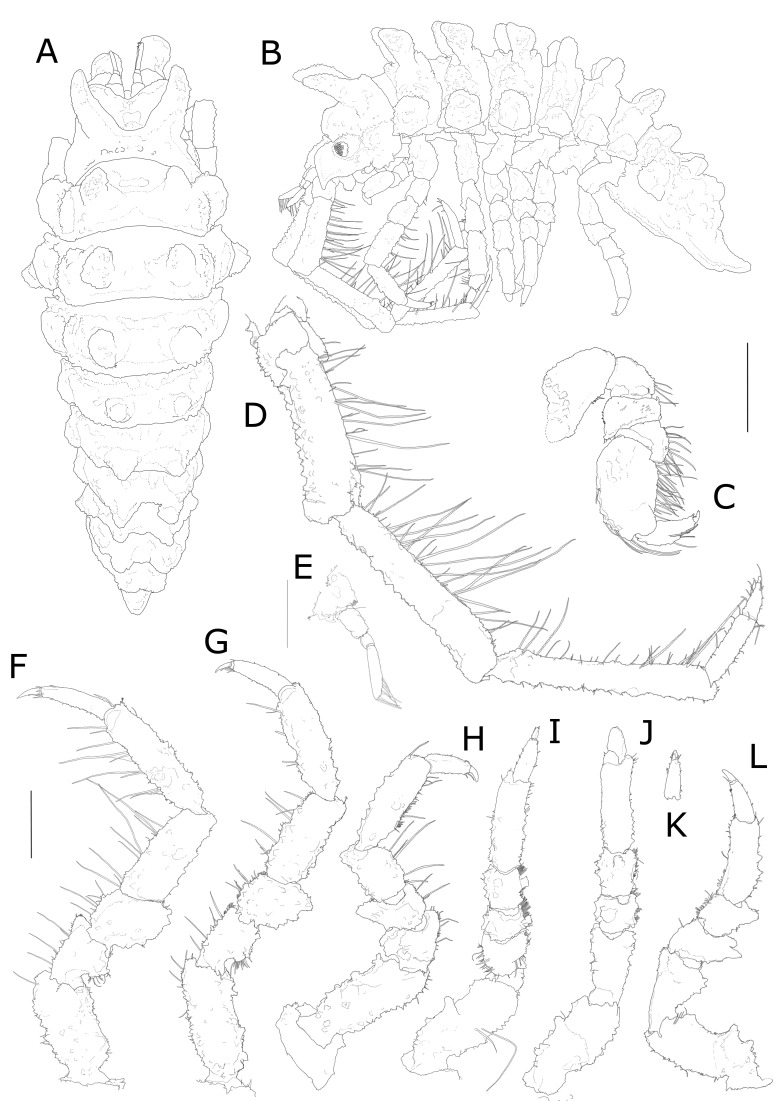
*Pseudidotheaarmata* sp. n. male holotype (MNA 10749). **A** dorsal view; **B** lateral view; **C** P1. Scale refers to 0.5 mm length. **D** A2; **E** A1; **F** P2; **G** P3; **H** P4; **I** P5; **J** P6; **K** Dactylus of P6; **L** P7. Scale refers to 1 mm length for **D-L**.

**Figure 3.**
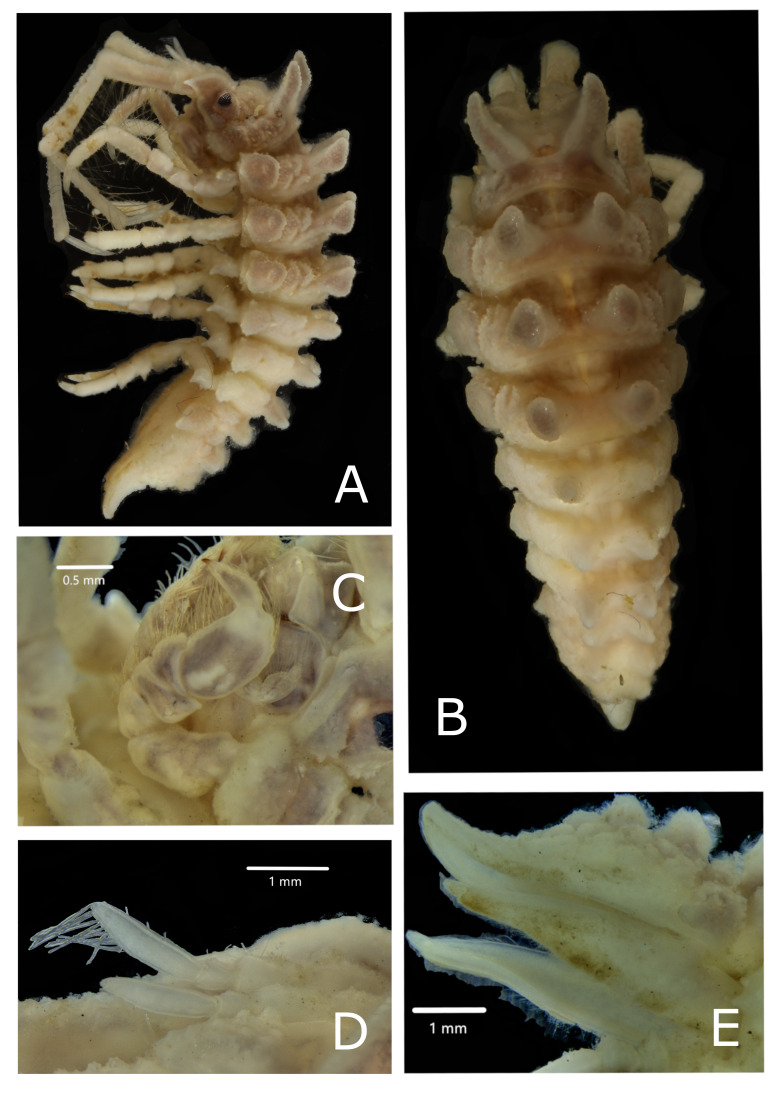
*Pseudidotheaarmata* sp. n. male holotype (MNA 10749). **A** stack photo of lateral view; **B** stack photo of dorsal view; **C** stack photo of P1, scale bar represents 0.5 mm; **D** stack photo of A1, scale bar represents 1 mm; **E** stack photo of pleotelson. Scale bar represents 1 mm length.

**Body**: Entire body surface rough and granular, covered with small hair-like setae (Fig. 2A, B and Fig. 3A, B).

CEPHALOTHORAX. Head with two frontomedial lobes with many small tubercle-like protrusions. Cephalothorax with two large and stout spines, frontally directed (Fig. 2A, B and Fig. 3A, B, Suppl. materials 1, 2), covered with tubercles and with diffused short hair-like setae (not illustrated in drawings, but visible in Fig. 3). Two lateral eyes of medium size, slightly more subtriangular than oval (Fig. 2A B and Fig. 3A, B, Suppl. material 1).

ANTENNA 1. First Antenna (A1) consisting of three peduncular and two flagellar articles. First peduncular article broadest, almost surpassing in width the length of the second peducular article, with one mediolateral simple bristle; second peduncular article long, nearly 1.5 times the first, the third almost a third of the second, rounded distally. First flagellar article short, ring-like, barely distinguishable; last flagellar article as long as the length of all the other articles of the A1. Distoventrally on this article, six pairs of aesthetascs, another single aesthetasc present in the tip of the A1 together with two simple setae (Fig. 2E and Fig. 3D).

ANTENNA 2. Second Antenna (A2) half as long as body, consisting of five peduncular and three flagellar articles. First peduncular article very small, second about double in length of the first; third peduncular article 3 times longer than second, with a two ventral rows of long simple setae, each group with one long and one short seta. Fourth and fifth peduncular articles longest, nearly subequal in length, with similar setae pattern of the third article. Last peduncular article with a distolateral small feather-like seta. First flagellar article about twice as long as second and third flagellar articles together, bearing short bristles and one longer apical simple seta. Second article smaller and narrower than first, with many simple short bristles. Last flagellar article smallest, claw-like. The whole antenna covered with lots of short and small hairs (Fig. 2D).

PEREONITES. Pereonite 1 fused with cephalothorax, but separated by a ridge. Pereonite 2 only slightly shorther than pereonite 3; pereonite 4 longest. Pereonite 5 slightly shorter than pereonite 2, pereonite 6 and 7 smallest and shortest. Pereonite 1 with one pair of large dorsal spine-like tubercles, anteriorly directed. Pereonites 2-4 with two pairs of tubercles (one spine-like dorsal pair and one shield-like lateral pair). Dorsal pair of tubercles in pereonites 2-4 are large and high, apically flattened, covered with few hair-like small setae and small tubercle-like protrusions; in pereonites 5-7, these are smaller, more slender, apically acute, but with blunt tip and more laterally flattened. Lateral pairs of tubercles are large in pereonites 2-4, smaller in pereonites 5-7. Evident elevations separated by deep grooves characterise the surface of pereonites 2-4, between the dorsal and lateral tubercles. Elevations are also present on pereonites 5-7. However, these are more shallow, barely visible and uniformly covering the surface between the dorsal and lateral tubercles (Fig. 2A, B and Fig. 3A, B).

PLEOTELSON. All pleonites fused to pleotelson, frontolaterally of pleotelson two stout and slightly rounded protrusions, caudally directed (Fig. 2A, B and Fig. 3E). Pleotelson frontally broadest, narrowing caudally. Tip of telson acuminating, but with blunt tip, slightly rounded, slightly bent dorsally; subapical telsonic spines absent, but two pairs of rounded large tubercles in two rows in the pleotelson, dorsally; the pleotelson lacks spines, but is covered entirely by medium-sized rounded protrusions, slightly smaller laterally (Fig. 2A, B and Fig. 3E).

**Figure 4.**
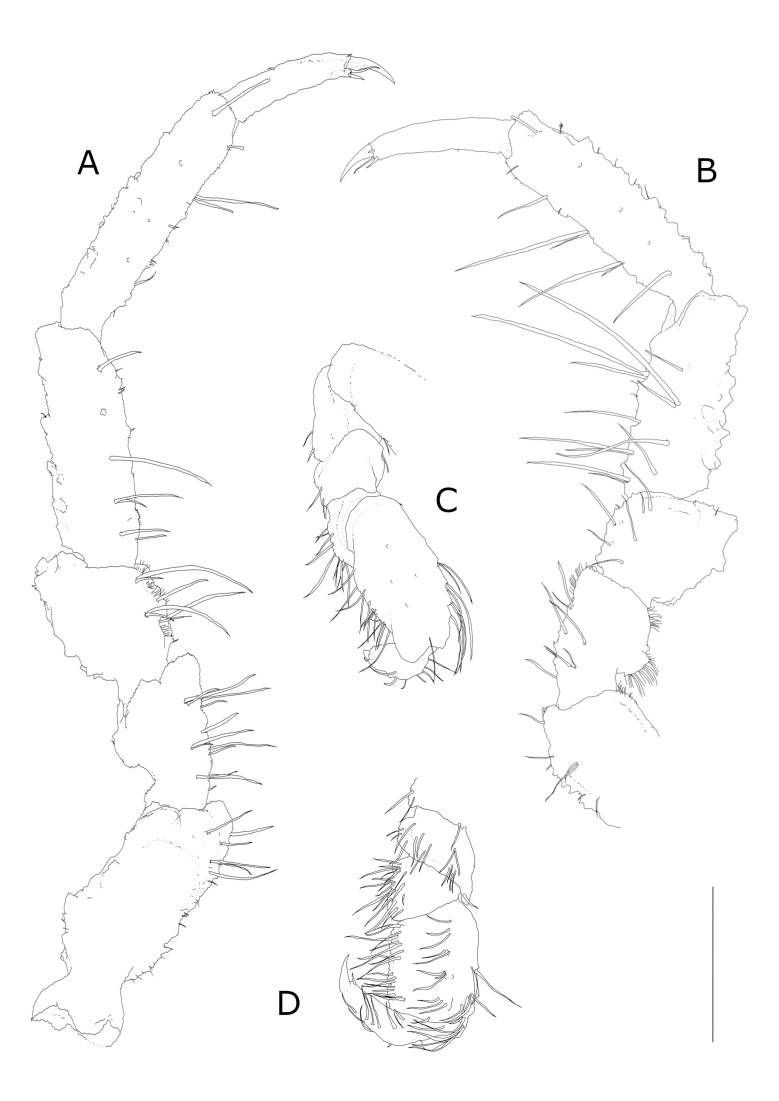
*Pseudidotheaarmata* sp. n. male holotype (MNA 10749). **A** left P2; **B** right P2; **C** right P1, dorsal part; **D** right P1, focus on inner part. Scale refers to 1 mm length.

PEREOPODS. P1 shorther than P2-7. Basis and propodus longest; basis with few distoventral and distolateral simple setae; a row of small protuberances on the lateral outer-directed side of the article. Carpus trapezoidal, ischium, merus and carpus densely covered with sensory spines, especially on ventral and lateral surfaces. Subchelate propodus broad, oval, dactylus shorter than propodus, with two short smooth claws (the ventral one shorter) and a small spine in between (Fig. 4C). Ventral surface of propodus forming slightly concave “spoon”, medial surface with few short simple bristles (Fig. 2C, Fig. 3C and Fig. 4D). P2-4 similar, one strong spine dorsolaterally on basis of P2-P4, distodorsal margins of ischium, carpus and propodus without strong spines, but with rough cuticular surface; merus of P2-4 presents an evident protrusion on the dorsal surface of the distal part; important protrusion is also present dorsomedially on ischium of P2 and P4. Setation in P2-4 is similar, but most prominent in P2. Merus, carpus and propodus with groups of setae arranged in two ventral rows, each group consisting of one long seta and one short seta (Fig. 4A and B). Small hairs present in all pereopods (Fig. 2F, G, H and Fig. 4A, B). P5-7 slightly shorter, but subequal in length to P2-4. P5 with two long setae on basis, P6 and 7 without long setae. Basis of P5-7 presenting one stout rounded spine dorsolaterally, in addition to smaller spines; stout spines are also present on carpus and merus (Fig. 2I, J, L and K). In P5-7, basis longest, ischium and propodus elongated compared to other articles; dactylus presenting a terminal claw with one simple seta in the terminal part. On propodus of P5-7, some feather-like bristles; all pereopods covered with small hairs (Fig. 2I, J and L).

UROPODS. Ventral surface of uropods covered with many simple hairs. Uropod elongated, both rami of uropods about subequal in length (Fig. 3E).

#### Diagnosis

The new species is characterised by one pair of large blunt anteriorly directed supra-ocular spines on the first pereonite. In dorsal view, these are long, divergent, forming a v-shape, reaching beyond the eyes. Pereonites 2-7 with only two pairs of tubercles: one dorsal pair of spine-like tubercles and one lateral pair of shield-like tubercles. Dorsal tubercles on pereonites 2-4 are large, apically flattened in lateral view, slightly anteriorly directed; on pereonites 5-7, these are smaller, slender, more pointed, but apically blunt and upwards directed. Pleotelson with blunt, strong protrusions.

#### Etymology

The species is named *armata* for its long supra-ocular and the dorso-apical spine-like tubercles, not simple “shielded” tubercles like *Pseudidotheascutata*, but more like strong blunt “spines”: as a contraposition to *P.scutata* meaning “that bears shield”, *P.armata* sp. n. “bears weapons”.

#### Distribution

Only known from type locality, the Burdwood Bank (Antarctica), found at 852 m.

#### Conservation

Specimen is stored and fixed with 96% ethanol in order to preserve it for further genetic analysis.

#### Remarks

*Pseudidothea* Ohlin, 1901 is clearly distinguishable from other genera by the oval shape of the body and peculiar conformation of pereonites and first pereopod. *P.bonnieri* was the type species representing the genus, described by Ohlin (1901). Subsequently, it was synonymised with *P.miersi* (Studer 1884) because of the many similarities with the latter species. The genus is only known from the Southern Hemisphere and it is mostly known from the Antarctic Peninsula in the Southern Ocean. A record of *Pseudidothea* Ohlin, 1901 was also recorded in the Ross Sea (Fig. 1), although undetermined to species level (Mackay 2018). To date, four species belong to the genus *Pseudidothea* as referred to above (original names are presented; the type species is marked with an asterisk):

*Pseudidotheahoplites* - Poore and Bardsley (2004)

*Pseudidotheamiersi - Ohlin 1901

*Pseudidothearichardsoni* - Hurley 1957

*Pseudidotheascutata* - Stephensen 1947

*Pseudidotheaarmata* sp. n.

#### Differential diagnosis

Within the genus *Pseudidothea*, *P.scutata* (Stephensen 1947) is most similar to *P.armata* sp. n. in shape and spine pattern. The main differences are the large supra-ocular spines and general body armature, that significantly differs from the apically flattened tubercles in *P.scutata*; similar is also the position of the flattened tubercles dorsally located in every pereonite of *P.scutata*; however, all of these structures are more like blunt spines in *P.armata* sp. n. Another difference can be found in the extreme reduction of the elevations present in between lateral flattened tubercles and dorsal spine-like tubercles. Brandt and Wägele (1990) re-described *P.scutata* and illustrated flattened, irregular elevations on pereonites 5-7, while in *P.armata* sp. n., these are barely present. The pleotelson of *P.scutata* and *P.armata* sp. n. is similar in shape and tubercular pattern; however, it completely lacks pleotelsonic dorsal spines in *P.armata*, only rounded, short and strong tubercles are present in the latter species.

Supra-ocular spines of *P.armata* are long, dorsally pointed and anteriorly directed, reaching beyond the eyes in dorsal view. *P.scutata* presents supraocular tubercles that are shorter, dorsally flattened and do not reach the eyes on dorsal view. In addition, supra-ocular spines in *P.armata* are divergent, forming a v-shape in dorsal view, while in *P.scutata* the supra-ocular tubercles are aligned parallel. The body armature in the two species shows a different pattern of tubercles. In *P.armata*, dorsal tubercles are more similar to blunt spines, apically less flattened than those present in *P.scutata*. In particular, *P.armata*'s spine-like tubercles on pereonites 5-7 are not apically flattened at all, but slender and pointed with a blunt apex, while in *P.armata*, pereonites 5-7 have small apically flat tubercles. *Pseudidotheascutata* presents raised areas in the lateral position, between the dorsal and lateral tubercles; these are marked on pereonites 5-7 and even described as distinct tubercles (Brandt and Wägele 1990, White 1992). *Pseudidotheaarmata* presents raised areas as well. However, elevations are more evident on pereonites 2-4, separated by deep grooves, making the surface between the dorsal and lateral tubercles look “wrinkly”, while elevations on pereonites 5-7 are less visible. Basis of P5 in *P.armata* has two long setae, while there are only simple setae on P5 of *P.scutata*.

The pleotelson of *P.scutata* and *P.armata* is similar in shape and tubercular pattern; however, it completely lacks pleotelsonic dorsal spines in *P.armata*; only rounded, short and strong tubercles are present in the latter species.

#### Distribution

Only known from type locality.

## Identification Keys

### *Pseudidothea* Ohlin, 1901

**Table:** 

1	Pereonites 2 and 3 with forked dorsolateral spines; all pereonites with lateral rows of blade-like ridges, each with anteriorly and posteriorly directed spines; tergites produced laterally over coxae to form a shield with 3 points	*Pseudidotheahoplites*
–	Pereonites with low or high flat tubercles; tergites produced laterally as large tubercles or rounded or flattened laterally	2
2	Pereon with large high flat tubercles; pereonite 1 with dorsal pair, pereonites 2–4 with dorsal and lateral pair and pereonites 5–7 with dorsal, dorsolateral and lateral pair; male pleopod 2 with appendix masculina twice as long as rami	*Pseudidotheascutata*
–	Pereonites with high tubercles, laterally flattened; rounded, short and strong tubercles	3
3	Pereonites with strong laterally flattened tubercles; supra-ocular spines dorsally pointed and anteriorly directed, reaching beyond the eyes, forming a “v” shape in dorsal view; pereonites 5-7 with small apically flat tubercles	*Pseudidotheaarmata* sp. n.
–	Pereon with low irregular tubercles; male pleopod 2 with appendix masculina and rami subequal	4
4	Uropodal exopod with a single strong setae, endopod with 3 pappose setae; antenna 2 peduncle with long fine setae on articles 3–5; pereopods without tubercles; male pleopod 1 endopod with 5 lateral spinules proximally, 5 apical plumose setae; exopod with 15 spinules on lateral margin, tapering distally to an obtuse apex	*Pseudidothearichardsoni*
–	Uropod rami each with single seta; antenna 2 peduncle with short setae on articles 3–5; pereopods with tubercles; male pleopod 1 endopod with plumose setae marginally; exopod with 16–17 spinules laterally, with acute apex bent outwards	*Pseudidotheamiersii*

This key to species is based to the *Pseudidothea* key present in the work of Poore and Bardsley "Pseudidotheidae (Crustacea: Isopoda: Valvifera) reviewed with description of a new species, first from Australia" (Poore and Bardsley 2004).

## Supplementary Material

XML Treatment for Pseudidothea armata

287A7163-E8FF-5B7C-AE5A-EC9E8A0153BE 10.3897/BDJ.10.e76864.suppl1 Supplementary material 1 MNA 10749 LATERAL Data type Digital inking drawing Brief description *Pseudidotheaarmata* sp. n. male holotype (MNA 10749) lateral view. File: oo_617277.tif https://binary.pensoft.net/file/617277 NIcholas Noli

A3341359-3195-5B3D-B019-0DF4417699C3 10.3897/BDJ.10.e76864.suppl2 Supplementary material 2 MNA 10749 DORSAL Data type Digital inking drawing Brief description *Pseudidotheaarmata* sp. n. male holotype (MNA 10749) dorsal view. File: oo_617278.tif https://binary.pensoft.net/file/617278 Nicholas Noli
