# Supplementary figures and images for: Pseudidotheaarmata sp. n., a new isopod of the genus Pseudidothea (Crustacea, Malacostraca, Isopoda) from the Atlantic sector of the Southern Ocean
Source: Biodivers Data J. 2022 Feb 17;10:e76864. doi: 10.3897/BDJ.10.e76864 (PMC8873171; doi:10.3897/BDJ.10.e76864)

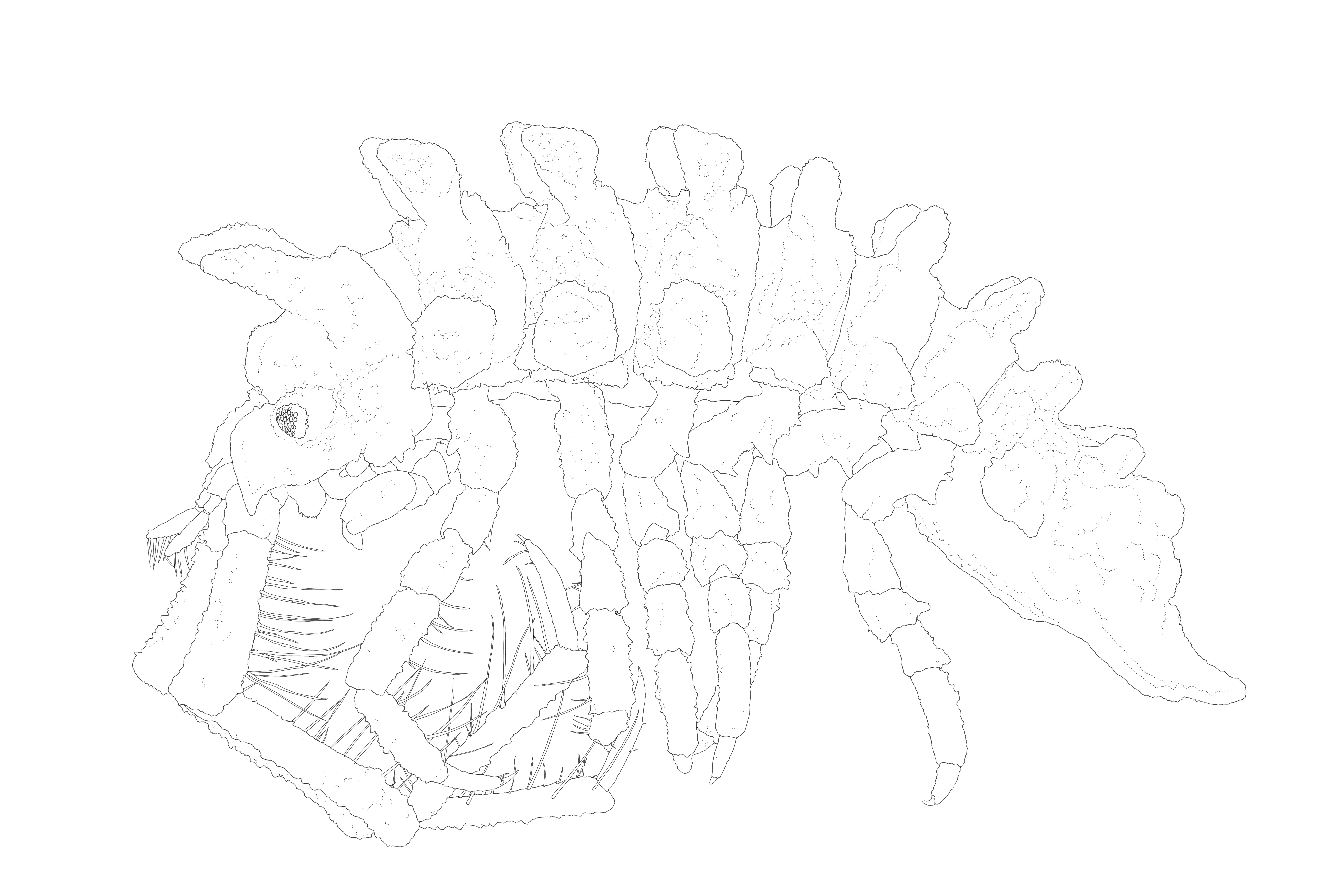

Supplement: Supplementary material 1 — MNA 10749 LATERAL [file bdj-10-e76864-s001.tif]

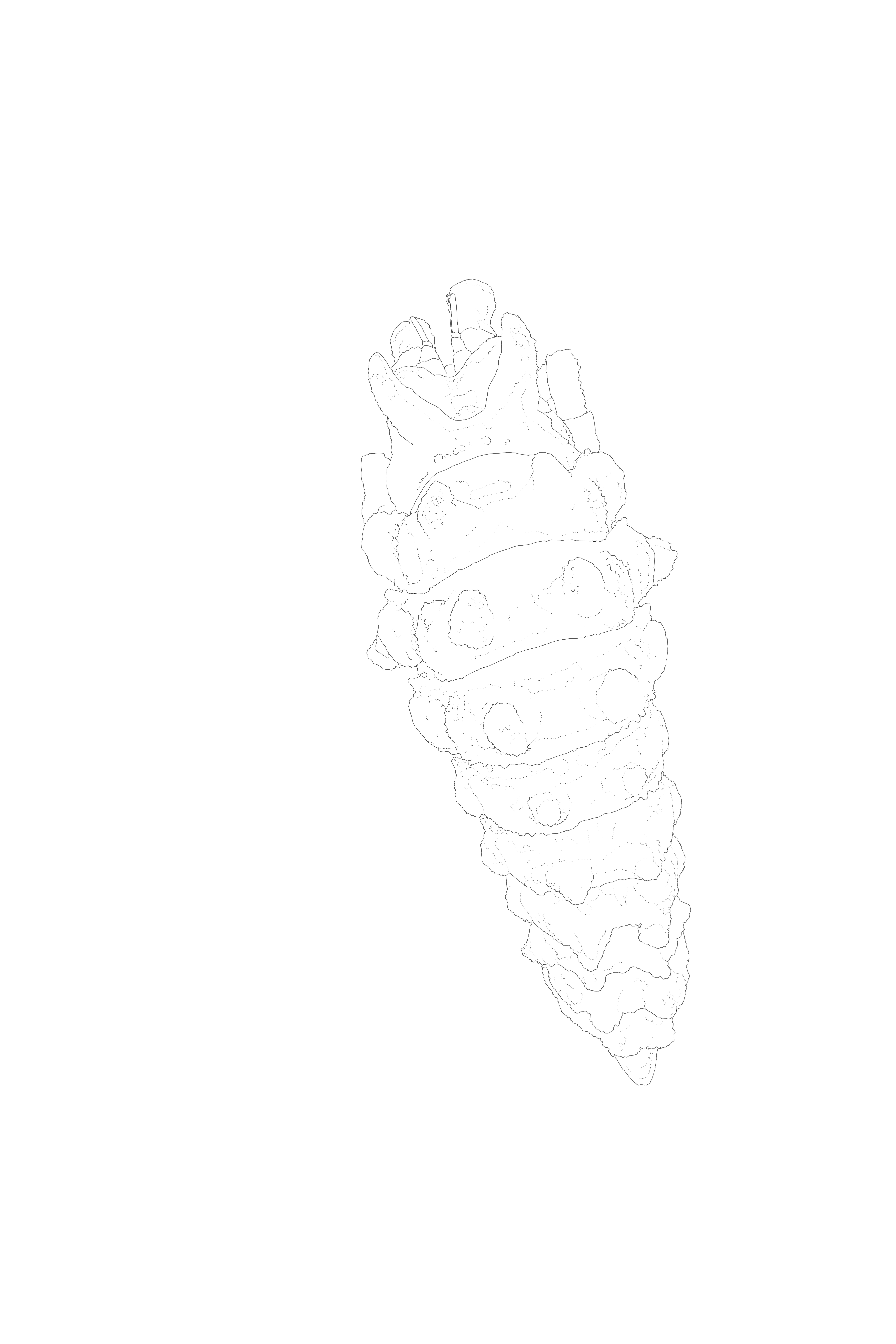

Supplement: Supplementary material 2 — MNA 10749 DORSAL [file bdj-10-e76864-s002.tif]
